# Reply to Yu‑Hsiang Lin, Chen‑Pang Hou, and Chun‑Te Wu’s Letter to the Editor Beyond traditional stenting: evaluating pigtail suture stents for reduced ureterorenoscopy symptoms. World J Urol 42, 255 (2024)

**DOI:** 10.1007/s00345-024-05033-x

**Published:** 2024-05-15

**Authors:** Benoît Vogt

**Affiliations:** Department of Urology, Polyclinique de Blois, 1 rue Robert Debré, 41260 La Chaussée Saint-Victor, France

**Keywords:** Renal stone, Ureteral stone, Ureteral stent, Stent failure, Stent-related symptoms, Malignant ureteral obstruction

We read with great interest the letter by Yu‑Hsiang Lin et al. regarding the randomized trials by Bosio et al. [1, 2]. These articles demonstrate that pigtail-suture stents (PSSs, JFil, Rocamed, Monaco) are better tolerated than double-pigtail stents (DPSs) in terms of stent-related symptoms (SRS) after ureteroscopy (URS). We would like to respond to the comments raised by the authors.

First, we thank Lin et al. for observing that PSSs, for which the sutures do not have an internal channel, do not alter the anti-reflux function of the ureterovesical junction. On the other hand, these sutures may be ineffective in draining urine through an edematous or tumor obstacle because the urine is then forced to pass between the sutures and the walls of the obstacle. The authors are right to point out this limitation, even if good clinical tolerance and respect for the ureterovesical junction are beneficial for the patient's quality of life. However, PSSs were initially designed so that the tube would contact the obstruction and the sutures would extend into a completely healthy underlying ureter. For example, the drainage of a lumbar stone by a PSS without any other procedure is a major indication. In this case, the sutures of the PSS limit SRS and subsequently allow easier URS by ureteral dilation [3].

Second, Bosio et al. should be congratulated for having the idea of using a PSS after an uncomplicated URS, with or without an access sheath and without pre-stenting. The 2021 study demonstrated that the tolerance of PSSs on D2 was greater than that of DPSs [1]. Thus, despite the limited effectiveness of sutures in treating possible ureteral edema, the absence of reflux and the reduction of intravesical material, specific to PSSs, have shown superiority in terms of tolerance. Bosio et al. thus offer the urologist an intermediate drainage solution after URS. Between the absence of drainage [4, 5] and drainage by a DPS [5], the urologist can drain using a PSS while knowing that the management of temporary pain is better experienced by the patient than with a DPS lasting 1 or 2 weeks [1–3].

Finally, Lin et al. suggest that vesicoureteral reflux induced by tumor stents can alter therapeutic management in cases of ureteral obstruction by the tumor. In this case, a PSS would be indicated but it is not recommended in its current version for the drainage of tumors, even if the tube is in contact with the obstruction. Indeed, previous studies have determined that the marketed PSS tube may be insufficiently stiff in the face of tumor compression [6]. In the event of tube compression, loss of the internal lumen can lead to drainage failure, resulting in infections or renal failure [6–8]. Ureteral obstruction and stent intolerance are frequent events that occur during treatment for obstructive tumors. Modification of the PSS into a tumor-PSS, as well as the availability of various other forms of stents adapted for compression, would allow more effective management of these patients [6, 8, 9].

## Data Availability

This Letter to the Editor is based on previously published literature and does not involve any new data collected by the authors. All analyses and discussions are derived from publicly available sources, which are cited within the manuscript. Therefore, there are no data sets generated or analyzed during the current study.
